# Adeno-associated virus serotypes 9 and rh10 mediate strong neuronal transduction of the dog brain

**DOI:** 10.1038/gt.2013.54

**Published:** 2013-10-17

**Authors:** G P Swain, M Prociuk, J H Bagel, P O'Donnell, K Berger, K Drobatz, B L Gurda, M E Haskins, M S Sands, C H Vite

**Affiliations:** 1 Departments of Clinical Studies and Pathobiology, School of Veterinary Medicine, University of Pennsylvania, Philadelphia, PA, USA; 2 Departments of Internal Medicine and Genetics, Washington University School of Medicine, St Louis, MO, USA

**Keywords:** animal model, AAV, canine, CNS

## Abstract

Canine models have many advantages for evaluating therapy of human central nervous system (CNS) diseases. In contrast to nonhuman primate models, naturally occurring canine CNS diseases are common. In contrast to murine models, the dog's lifespan is long, its brain is large and the diseases affecting it commonly have the same molecular, pathological and clinical phenotype as the human diseases. We compared the ability of four intracerebrally injected adeno-associated virus vector (AAV) serotypes to transduce the dog brain with green fluorescent protein as the first step in using these vectors to evaluate both delivery and efficacy in naturally occurring canine homologs of human diseases. Quantitative measures of transduction, maximum diameter and area, identified both AAV2/9 and AAV2/rh10 as significantly more efficient than either AAV2/1 or AAV2/5 at transducing cerebral cortex, caudate nucleus, thalamus and internal capsule. Fluorescence co-labeling with cell-type-specific antibodies demonstrated that AAV2/9 and AAV2/rh10 were capable of primarily transducing neurons, although glial transduction was also identified and found to be more efficient with the AAV2/9 vector. These data are a prerequisite to evaluating the efficacy of recombinant AAV vectors carrying disease-modifying transgenes to treat naturally occurring canine models in preclinical studies of human CNS disease therapy.

## Introduction

A large number of naturally occurring central nervous system (CNS) diseases due to specific single gene defects have been identified in dogs, including 19 lysosomal storage diseases due to mutations in 26 genes (Table 1).^1, 2, 3, 4, 5, 6, 7, 8, 9, 10, 11, 12, 13, 14, 15^ These canine models exhibit the molecular, biochemical, pathological and clinical abnormalities seen in patients making them true homologs of human disease. Canine models have several advantages over other animal models of human CNS disease. First, in contrast to nonhuman primate (NHP) models that are rare,^16, 17^ naturally occurring CNS diseases in the dog are common.^18, 19, 20, 21, 22, 23, 24, 25, 26, 27, 28, 29, 30, 31^ Second, in contrast to murine models, the large size and physical organization of the dog brain is more similar to the human brain,^30^ and the background genetic heterogeneity among relatively non-inbred dogs is similar to the genetic diversity of affected human populations.^31^ Finally, the lifespan of dogs permits long-term evaluation of the safety and efficacy of experimental therapies. Each of these advantages justifies the evaluation of CNS disease therapy in canine models.

Although canine models of CNS disease are powerful tools for preclinical studies, to date few gene therapy studies have been performed, and it is not known which adeno-associated virus vector (AAV) serotypes efficiently transduce the canine brain. However, it is clear that specific AAV capsid protein domains can mediate different levels of transduction in different species.^32, 33, 34, 35, 36^ Therefore, we evaluated the ability of four AAV serotypes (AAV1, 5, 9 and rh10) to transduce the normal dog brain with a reporter gene. These serotypes were chosen because of their ability to transduce the CNS of several species relatively efficiently.^35, 36, 37, 38, 39^ Brain regions injected included cerebral cortex, subcortical gray matter and white matter, regions that are commonly affected in neurodegenerative diseases. These data are a prerequisite to evaluating the efficacy of recombinant AAV vector (rAAV) carrying disease-modifying transgenes to treat naturally occurring canine models of human CNS diseases.^40^

## Results and Discussion

**Figure 1:**
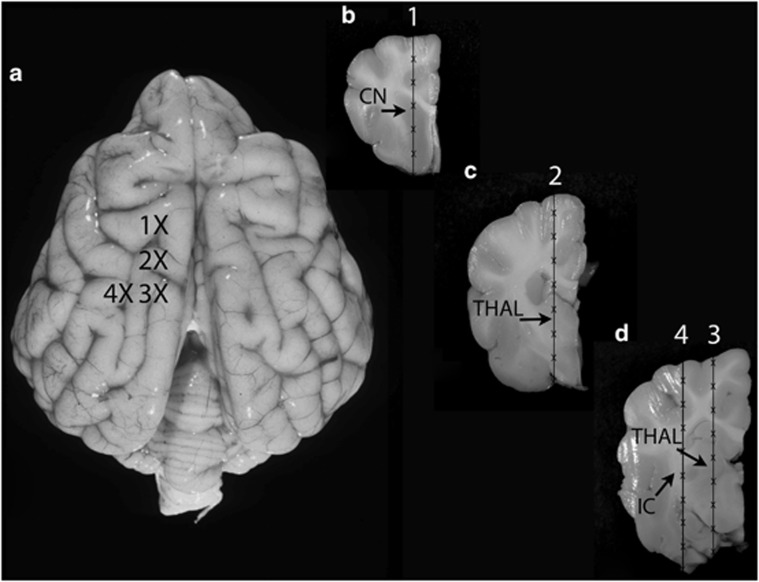
Injection sites in the dog brain. A dorsal plane image (**a**) and transverse images (**b–d**) of the dog brain are shown. The injection sites in the left cerebral hemisphere are identified by Xs. A Hamilton syringe and needle were used to deliver each serotype to the brain. Each dog was injected with one rAAV serotype, and each serotype was injected into two dogs. Transduction of the caudate nucleus (CN) was assessed at site 1 (**b**); transduction of the thalamus (THAL) was assessed at sites 2 (**c**) and 3 (**d**), and transduction of the white matter (internal capsule (IC)) was assessed at site 4 (**d**).

We compared the ability of four distinct AAV serotypes containing vector genome encoding for green fluorescent protein (GFP) under the control of the CAGS promoter to transduce cells in the normal dog brain. Each of the four recombinant vectors, designated AAV2/1, AAV2/5, AAV2/9 and AAV2/rh10, contained the same genome with the only differences being the inherent amino-acid composition of the capsids and the concentrations of vector (see Materials and methods); the total genome copies (GC) delivered per needle track was 5.13 × 10^11^ GC, 7.02 × 10^11^ GC, 1.66 × 10^11^ GC and 2.54 × 10^11^ GC for AAV2/1, AAV2/5, AAV2/9 and AAV2/rh10, respectively, making the final delivered doses of AAV5>1>rh10>9. The left cerebral hemisphere of 12–16-week-old dogs was intracerebrally injected along four vertically oriented columns with 3 ul injected every 0.5 cm (Figure 1). Each serotype was injected into the brain of two dogs. All dogs recovered and were eating well within 12 h of surgery. No dogs showed any abnormal clinical signs during the 5-week observation period after which time they were euthanized.

**Figure 2:**
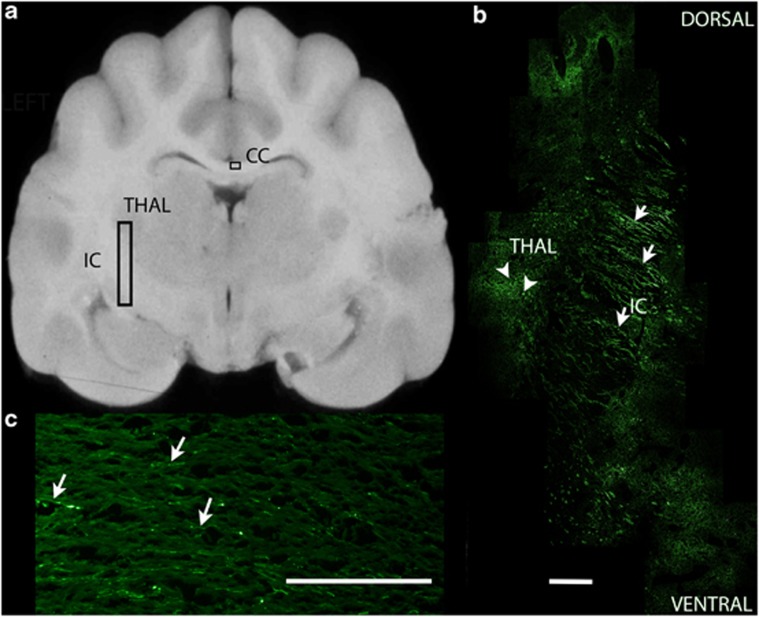
GFP fluorescence in a dog brain injected with AAV2/9. (**a**) A transverse section of the dog brain is shown at the level of injection site 4 in the left cerebral hemisphere. The vertical rectangle shows the region of brain photographed in **b**. The smaller horizontal rectangle shows the region photographed in **c**. (**b**) Transduction of somata (arrowheads) characterized by large punctate areas of the thalamus (THAL), and fluorescence of white matter axons (arrows) characterized by linear regions of the internal capsule are present. Dorsal is at the top of the image and ventral at the bottom. The scale bar for the photomicrograph is 1 mm. (**c**) GFP fluorescence in fibers of the corpus callosum of the right cerebral hemisphere. Fluorescent axons (arrows) were seen extending from the white matter of the injected hemisphere, crossing the corpus callosum (CC) and entering the contralateral uninjected hemisphere.

The large size of the brain required that each brain be divided into ∼30 blocks for embedding. Transverse brain sections from each block were first evaluated in a blind fashion with the observer using a semiquantitative scale of 0–4 to grade the fluorescence of cell somata in three gray matter regions at the site of an injection track in each dog (caudate nucleus, thalamus, cerebral cortex, Table 2), as well as fluorescence of axons in three white matter regions. AAV2/5 resulted in sparse fluorescent somata in the thalamus and cerebral cortex of one of the injected dogs (dog 3). Sparse axons, identified as such by their length and directionality, located in adjacent temporal lobe white matter, were also found to fluoresce. In the second dog injected with AAV2/5 (dog 4), sparse axons in the temporal lobe white matter were found to fluoresce, although no somata were identified even though over 100 slides from each of the tissue blocks were evaluated. It was assumed that a very limited number of cells were transduced in this dog. Sections evaluated from brain regions not including the thalamus showed no evidence of GFP in somata or axons in either dog. AAV2/1 resulted in subjectively more GFP-positive somata in the caudate nucleus, thalamus and cerebral cortex (dogs 1 and 2), as well as in axons of adjacent white matter. Fluorescent axons were found in multiple sections of brain and as far away from the injection track as the occipital lobe white matter in the left hemisphere. Both AAV2/9 (dogs 5 and 6) and AAV2/rh10 (dogs 7 and 8) showed extensive GFP expression in cell bodies of the caudate nucleus, thalamus, with subjectively less expression in the cerebral cortex (Figures 2a and b). Extensive GFP expression was also evident in the axons of the white matter of the temporal and occipital lobes (Figure 2b). Although no transduction of somata was noted in sections away from the injection track, fluorescent axons were again visible as far away as the occipital white matter (3 cm from the injection track). Interestingly, one dog injected with AAV2/9 and one with AAV2/rh10 showed GFP expression in the axons of the contralateral cerebral hemisphere with evidence of GFP-positive axons crossing the corpus callosum and also present in the contralateral corona radiata (Figure 2c) and internal capsule. No fluorescent somata were identified in any dog within the caudal brain stem or cerebellum. Finally, although the cerebral cortex was examined for the laminar distribution of GFP expression, cytoarchitectonics were difficult to evaluate in frozen sections. However, it was possible to identify that multiple cortical lamina showed GFP expression in AAV2/1-, 2/9- and rh10-injected brain.

**Figure 3:**
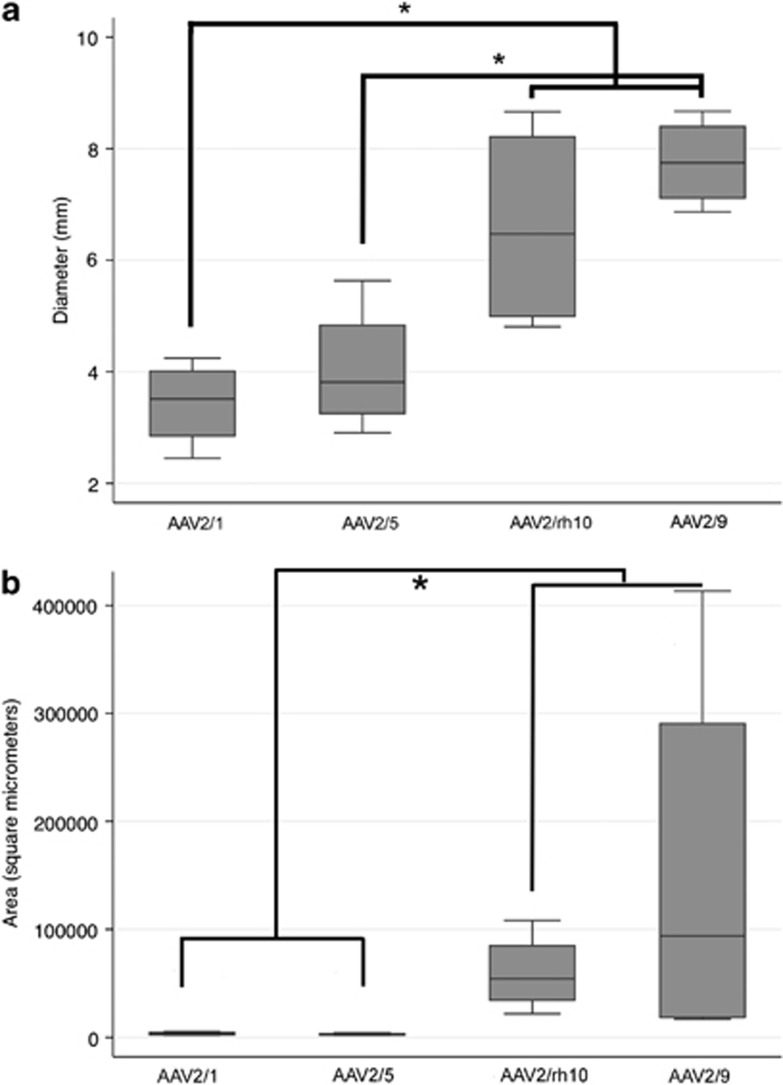
Boxplots showing the median, and 25th and 75th percentile of the data distribution for the maximum diameter (**a**) and maximum area (**b**) of transduction for each of the four rAAV serotypes. The Kruskal–Wallis test was used to compare data between groups and the Mann–Whitney test was used for pairwise comparison. *P<0.05. Significantly increased diameter was identified in both AAV2/9 and AAV2/rh10 compared with AAV2/1, whereas only AAV2/9 was significantly greater than AAV2/5. Significantly increased area of transduction was also identified in both AAV2/9 and AAV2/rh10 compared with either AAV2/1 or AAV2/5. Finally, AAV2/9 and AAV2/rh10 were not significantly different from each other in either maximum diameter or area.

Maximum mediolateral diameter as well as maximum area of fluorescence, including both somata and axons, were measured on two sections containing caudate nucleus and thalamus (injection sites 1 and 2) in each dog (*n*=4) for each serotype. As injection tracks could not be reliably identified in dog 4, only dog 3 had diameters and areas determined for AAV2/5. AAV2/1 and AAV2/5 showed similar maximum diameter and area of transduction (Figure 3). In contrast, both AAV2/9 and AAV2/rh10 showed diameter measurements significantly greater than AAV2/1, with AAV2/9 alone significantly greater than AAV2/5. No significant difference was identified between AAV2/9 and AAV2/rh10 in the maximum diameter of transduction (Figure 3a). Significantly greater maximum areas of transduction were identified for AAV2/9 and AAV2/rh10 compared with either AAV2/1 or AAV2/5, although large ranges were noted in the former two serotypes. In the two dogs injected with AAV2/9, the maximum area of transduction at injection site 2 was 413 339 μm^2^ in one dog and 20 167 μm^2^ in the second. In the two dogs injected with AAV2/rh10, the maximum area of transduction at injection site 2 was 108 217 μm^2^ in one dog and 21 881 μm^2^ in the second. It is important to note that although the dose of AAV2/9 was ∼4.2-fold lower than the dose of AAV2/5, it was significantly more efficient. In addition, although no significant difference between AAV2/9 and AAV2/rh10 could be identified (Figure 3b), one dog injected with AAV2/9 showed a maximum area of transduction almost four times greater than either dog injected with AAV2/rh10. Clearly, a larger number of dogs would be needed to evaluate whether AAV9 is superior to AAVrh10 in terms of area of transduction. Titer matching of AAV2/9 and AAV2/rh10, which was not performed because of a decision to evaluate the highest titer available of each vector for planned therapy experiments, would also be required for the most accurate comparison of these two serotypes.

Significant variability of transduction efficiency between brains injected with the same serotype was evident in our study (Figures 3a and b) and has been described by others.^35, 41^ Variability in regional transduction was also identified between dogs (Table 2). Although all vectors were capable of transducing cells of the cerebral cortex and thalamus, all dogs did not show the same fluorescence in these target regions. Specifically, AAV2/1, 2/9 and 2/rh10 were capable of transducing cells of the caudate nucleus in dogs 1, 5, 6 and 7, although GFP was not identified in this nucleus in dogs 2, 3 and 8. It is believed that slight differences in angle of the needle track were responsible for the failure of expression in these dogs, as both gray and white matter immediately caudal to the caudate nucleus were transduced in dogs 2, 3 and 8.

Evidence of spread of the transgene product to the contralateral hemisphere through axonal tracts has been described in mice.^35, 42, 43^ In the dog, AAV2/1, 2/9 and 2/rh10 each resulted in GFP fluorescence of axons of the white matter greater than 3 cm from the most caudal injection track. Both AAV2/9 and AAV2/rh10 also demonstrated GFP expression in the axons of the contralateral corpus callosum. These data demonstrate the ability of these vectors to express GFP in both neurons and axons, and indicate that axonal transport of transgene product could deliver protein to large areas of the brain using fewer therapeutic injections as has been proposed in mice.^42, 44^

**Figure 4:**
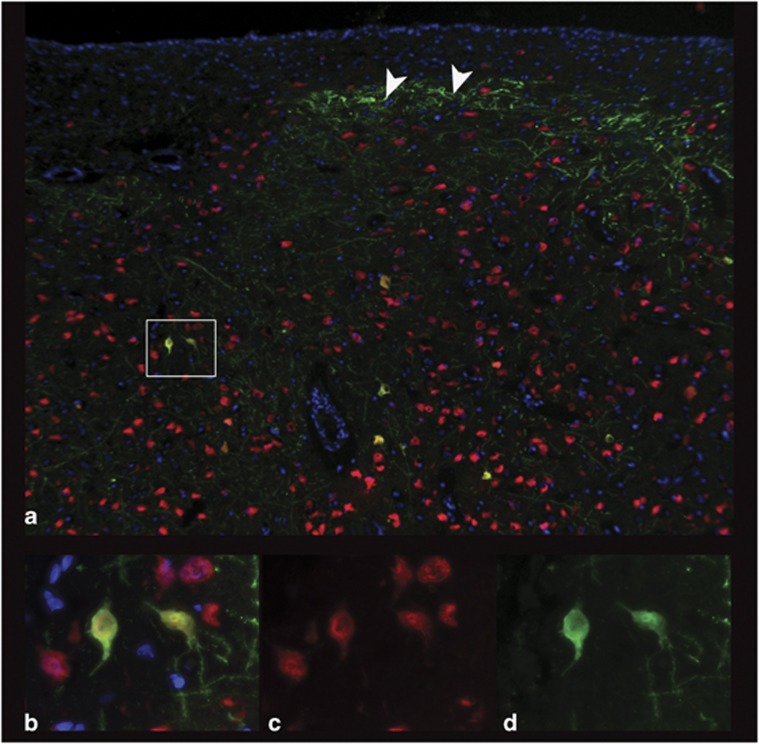
Neuronal transduction by AAV2/9. NeuN-positive neurons of the thalamus transduced by GFP in dog brain injected with AAV2/9 vector. (**a**) × 10 magnification showing GFP fluorescence (green), NeuN fluorescence (red), 4',6-diamidino-2-phenylindole (blue) and regions of co-labeling (orange). Rectangle shows × 100 magnification (**b**–**d**) with orange co-labeled neurons (**b**) red NeuN-positive cells alone (**c**) and GFP-positive cells alone (**d**). Arrowheads show astrocytes.

**Figure 5:**
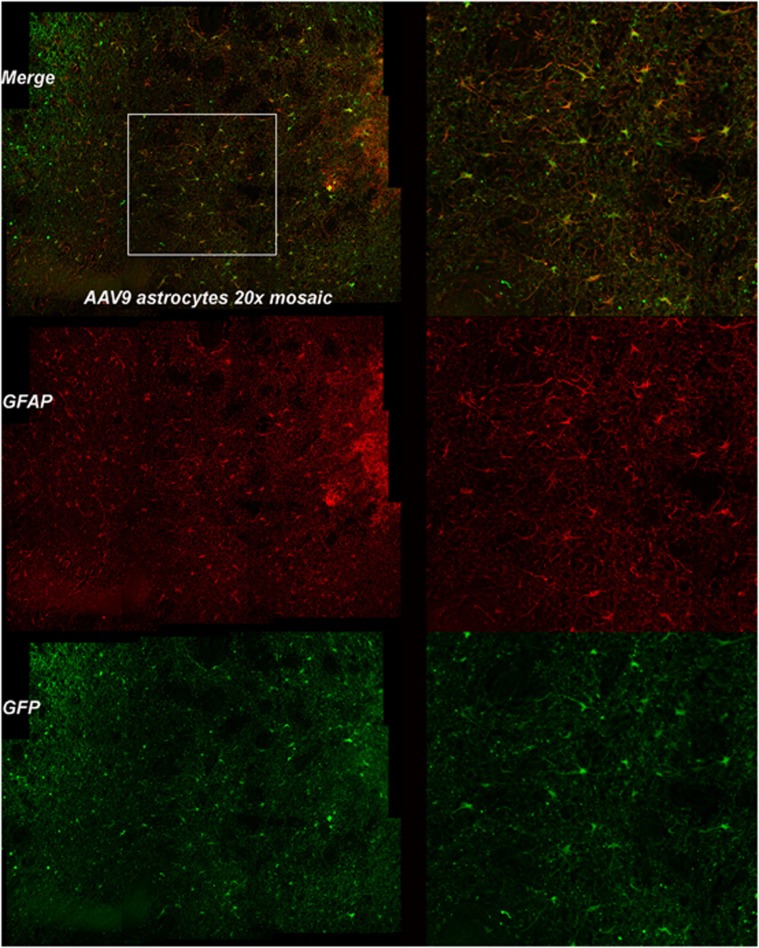
Astrocytic transduction by AAV2/9. GFAP-positive astrocytes of the caudate nucleus of the dog brain transduced with the AAV2/9 vector. Left column shows × 20 magnification showing GFP fluorescence (green), GFAP fluorescence (red) and regions of co-labeling (orange). Region within rectangle is present in second column and shows × 100 magnification with orange co-labeled astrocytes, red GFAP-positive cells alone and GFP-positive cells alone.

**Figure 6:**
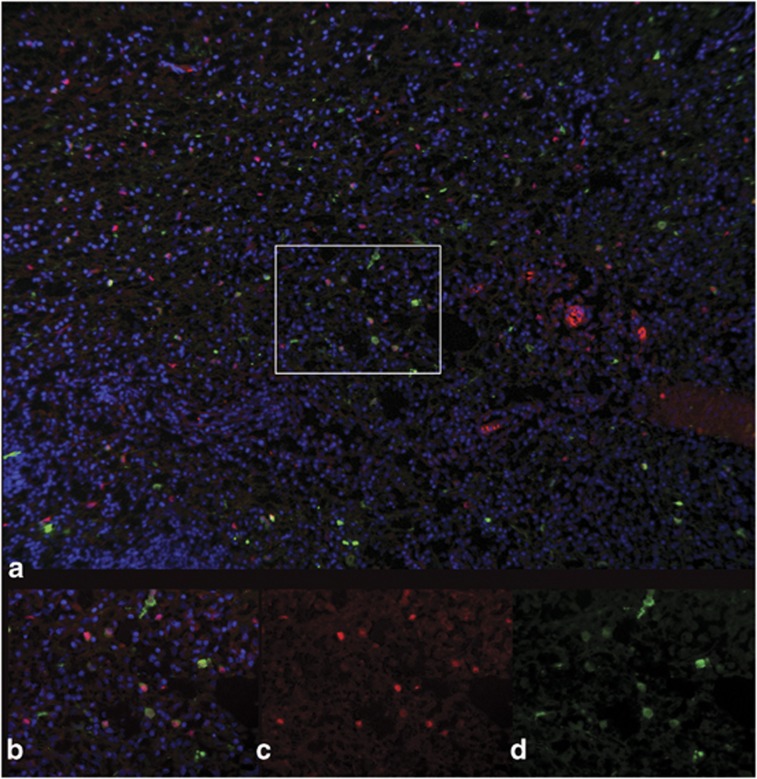
Oligodendrocytic transduction by GFP. GFP-positive, OLIG2-positive oligodendrocytes of the thalamus of the dog brain transduced with the AAV2/rh10 vector (**a**), × 10 magnification showing GFP fluorescence (green), nuclear OLIG2 fluorescence (red), 4',6-diamidino-2-phenylindole (blue) and regions of co-labeling (orange). Rectangle shows × 40 magnification (**b-d**) with co-labeled oligodendrocytes (**b**), OLIG2-positive cells alone (**c**) and GFP-positive cells alone (**d**).

Finally, to characterize the cellular tropism of each AAV serotype in the dog brain, fluorescence co-labeling with cell-type-specific antibodies was performed to evaluate GFP transduction by the three serotypes, AAV2/1, AAV2/9 and AAV2/rh10. Each of these three vectors strongly transduced neurons (NeuN-labeled; Figure 4) as was also evident from their ability to result in fluorescent axons. In contrast, subjective evaluation revealed AAV2/9 strongly transduced astrocytes, evaluated by co-labeling with GFAP (Figure 5). Neither of the other two serotypes transduced astrocytes to the same extent as seen with AAV2/9. Oligodendrocyte transduction, as determined by Olig2, was poor and was only seen to a small extent in dogs injected with AAV2/9 and AAV2/rh10 (Figure 6). No GFP expression was seen in cells labeled with GFAP or Olig2 in AAV2/5-injected brain, and most GFP-positive cells were NeuN positive in this dog.

Compared with what is described in cats and nonhuman primates (NHPs), transduction patterns in the dog are similar to what is published. Vite *et al.*^33^ evaluated the ability of three AAV serotypes to transduce the cat brain. A transcription unit containing the human GUSB complementary DNA under the control of the human GUSB promoter was cross-packaged into AAV1, AAV2 and AAV5 capsids that were injected into the cerebral cortex, caudate nucleus, thalamus and white matter of normal cats. Transgene expression was evaluated by *in situ* hybridization and through histochemistry. The AAV2 vector was capable of transducing cells in the gray matter, whereas the AAV1 vector resulted in greater transduction of the gray matter than AAV2 as well as transduction of unidentified cells within the white matter. AAV5 did not result in detectable transduction in the cat brain. AAV9 and rh10 were not evaluated.

In NHPs, Ciron *et al.*^45^ evaluated the ability of AAV1, 2 and 5 to transduce the macaque brain. A transcription unit containing the human alpha-iduronidase complementary DNA under the control of the mouse phophoglycerate kinase promoter was packaged into AAV1, 2 and 5 capsids and injected into the putamen and internal capsule. Vector diffusion was evaluated via quantitative PCR and vector copy-numbers per cell were compared. Diffusion throughout the brain was not significantly different among the three serotypes but AAV1 and AAV5 resulted in higher vector copy-numbers per cell than did AAV2. AAV1 was also used to deliver human acid sphingomyelinase complementary DNA under the control of a cytomegalovirus enhancer/chicken beta-actin promoter to cynomolgus monkey brain.^46^ Transduction efficiency was evaluated by immunohistochemistry and *in situ* hybridization and therapeutic enzyme levels were achieved in broad regions of brain aided in part by axonal transport of AAV, AAV movement through perivascular spaces and secretion of acid sphingomyelinase into the extracellular space.

The AAV5 serotype has been evaluated in the canine brain^47, 48^ where intracerebral delivery of the genes encoding alpha-L-iduronidase and alpha-*N*-acetyl-glucosaminidase in the canine models of mucopolysaccharidosis type I and IIIB ameliorated the accumulation of lysosomal storage product as well as neuropathology providing evidence that canine models could be used to evaluate the efficacy of CNS gene therapy. The data provided in this manuscript provide compelling evidence that either AAV2/9 or AAV2/rh10 could further improve transduction throughout the canine brain.

Few large animal studies have been published on transduction patterns of intracerebrally injected AAV9 or AAVrh10 vectors. In one study, Masamizu *et al.*^49^ identified a preference for neurons with low transduction of astrocytes or oligodendrocytes when AAV9 was injected into the striatum of NHPs. Other AAV9 studies in NHPs have focused on intracisternal (cisterna magna) and intrathecal (lumbar) injections. In these studies, regardless of volume or dosage, two different groups also identified a neuronal preference with low transduction efficiency in glial cells, along with substantial transduction of multiple structures, including the choroid plexus, ependymal cells of the ventricles and Purkinje cells of the cerebellum.^50, 51^ The most recent large animal study for AAV9 in the CNS looked at a therapeutic transgene for treatment of MPS IIIA. Here, Haurigot *et al.*^52^ identified strong transduction in similar regions of dogs injected intracisternally or intracerebroventricularly, supporting the use of this vector for treatment of a variety of CNS diseases. Data in large animal models for AAVrh10 are even sparser. Rodent studies have currently laid the foundation for a neuronal cell preference when injected into the striatum for this vector, similar to that seen for AAV9.^37^ The data presented in this report are among the first to provide insight on transduction patterns of AAVrh10 in a large animal model, although another recent study has presented detection of transduced cells within the cortex, white matter, deep gray matter of the striatum, thalamus, choroid plexus and spinal cord dorsal root ganglion when injected intracerebrally in NHPs; the details are currently unavailable.^53^ This vector has also been implicated as having potential for treating CNS disorders and as such, translation into humans and treatment for CNS diseases are being studied through intracerebral injections of AAVrh10 for Battens (NCT01414985 and NCT01161576, clinicaltrials.gov) and MLD (NCT01801709, clinicaltrials.gov) in the clinic.

Importantly, the authors believe that the only clearly predictive transduction pattern for human brain will be obtained from data from AAV-injected human brain. However, we also strongly believe that evaluating experimental therapies in naturally occurring large animal models with larger brains than mice, and naturally occurring homologous diseases not found in NHPs, will be critical for predicting therapeutic efficacy in human trials. This is most effectively demonstrated by the experience with a disease of inherited blindness, Leber's congenital amaurosis. Subretinal injections of an AAV2 vector expressing the RPE65 protein resulted in long-term (>10 years) visual improvements in the canine model of Leber's congenital amaurosis.^54^ A similar vector and approach were used in the successful AAV-mediated gene therapy clinical trial for Leber's congenital amaurosis.^55, 56^

The data presented above are a prerequisite for evaluating the efficacy of rAAVs carrying disease-modifying transgenes to treat naturally occurring canine models of human CNS diseases.^40^ These canine models of human disease offer the ability to test efficacy, delivery, as well as long-term safety in one model system making them unique models for use in preclinical trials.

## Materials and methods

### rAAV vector production

The vector genome encoded AAV2 ITRs flanking a transcription unit containing GFP under the control of the CAGS promoter (CB7 with chicken beta-actin intron (CI)). The plasmid was cross-packaged by the Gene Therapy Program of the University of Pennsylvania into AAV1, AAV5, AAV9 and AAVrh10 capsids resulting in four recombinant viral vectors (rAAV) each containing the same single-stranded AAV2-genome (AAV2/1.CB7.CI.EGFP.RBG, AAV2/5.CB7.CI.EGFP.RBG, AAV2/9.CB7.CI.EGFP.RBG and AAV2/rh10.CB7.CI.EGFP.RBG, respectively). Large-scale vector preparations were generated as previously described.^57^ AAV GC were acquired with real-time PCR using TaqMan (Applied Biosystems, Foster City, CA, USA) reagents and equipment. All primers and probes were engineered to detect elements specific to the packaged genome. SDS-polyacrylamide gel electrophoresis analysis (virion proteins comprise >90% of the detected proteins) and endotoxin assays (<5 endotoxin units per ml) were used for purity measurements (and dictated release criteria). The titer of each of the four viral vectors, from here on designated AAV2/1, AAV2/5, AAV2/9 and AAV2/rh10 was 1.9 × 10^13^, 2.6 × 10^13^, 6.14 × 10^12^ and 9.4 10^12^ GC per ml, respectively.

### Animals

Eight dogs were raised in the animal colony of the School of Veterinary Medicine of the University of Pennsylvania, under the National Institutes of Health and United States Department of Agriculture guidelines for the care and use of animals in research. The animals were housed at 21 °C with *ad libitum* food and water, 12-h light cycles, with 12–15 air changes per hour. At 12–16 weeks of age, dogs were preanesthetized with 0.1 mg kg^−1^ hydromorphine intramuscularly, 0.02 mg kg^−1^ atropine IV and anesthesia was induced with intravenous propofol (2–6 mg kg^−1^ to effect). Animals were intubated and anesthesia was maintained with isoflurane and placed in a stereotaxic head holder (David Kopf Instruments, Tujunga, CA, USA). The dogs' hair was clipped and the skin scrubbed for aseptic surgery. A 4-cm long skin incision was made in the dorsal midline of the skin over the skull. A 25-ga drill bit was used to drill a 1-mm hole in the left side of the skull at the following positions: 0.5 cm lateral to the bregma suture; 0.5 cm lateral and 0.5 cm caudal to the bregma suture; 0.5 cm lateral and 1 cm caudal to the bregma suture; and 1 cm lateral and 1 cm caudal to the bregma suture (Figure 1). These sites were chosen to evaluate transduction at the level of the caudate nucleus (1), thalamus (2 & 3) and temporal lobe white matter including the internal capsule (4). A 25-ul Hamilton syringe with a 29-ga needle was used to administer each rAAV vector. Each dog received one serotype and each serotype was administered to two dogs. The needle was placed into the brain to the ventral most limit, and 3 ul of vector was injected and allowed to diffuse for 3 min. The needle was withdrawn 0.5 cm, and the injection was repeated. The needle was withdrawn vertically 0.5 cm distances and the injection procedure repeated until the needle was no longer in the brain. The same procedure was completed for all four columns. Between 18 and 27 ul of vector were injected in each column. Subcutaneous skin closure was made in a continuous pattern using 2-0 polydioxanone suture. Subcuticular skin closure was made in a continuous pattern using 2.0 polydioxanone suture.

### Preparation of brain tissue

The eight injected dogs were euthanized 5 weeks after surgery using an intravenous overdose of barbiturate (Euthasol, Virbac Animal Health, Fort Worth, TX, USA) in accordance with the American Veterinary Medical Association guidelines. Immediately before euthanasia, 0.5 s ml heparin (1000 U ml^−1^) was administered intravenously. Following death, intracardiac perfusion with 750 ml of 0.9% cold saline was performed. Brains were removed, sectioned transversely at ∼5 mm thickness, fixed in 4% paraformaldehyde for 48 h and then transferred to 30% sucrose-PBS (phosphate-buffered saline) at 4 °C until the sections no longer floated. Brain sections were then placed in optimal cutting temperature media (Tissue-Tek; Sakura Finetechnical Co, Tokyo, Japan), frozen and cryosectioned transversely at a thickness of 7 μm. Ten tissue sections from each block were placed on slides, with 10 sections skipped between each to produce slides covering ∼770 μm of brain, and stored at −80 °C until staining was performed.

### GFP fluorescence microscopy

Ten sections from each block of each dog brain were evaluated by fluorescence microscopy to identify a needle track that was defined as the section containing the most fluorescent cells from the dorsal to the ventral surface of the brain. The number of fluorescent cell somata and fibers was semiquantified (0=none; 1=sparse (<10 GFP-positive somata/ × 10 field; <5% GFP-positive axons/ × 10 field); 2=few (10–40 GFP-positive somata/ × 10 field; 5–10% GFP-positive axons/ × 10 field); 3=moderate (40–100 GFP-positive somata/ × 10 field; 10–25% GFP-positive axons/ × 10 field); 4=extensive (>100 GFP-positive somata/ × 10 field; >25% GFP-positive axons/ × 10 field) on the needle track section. The observer was blinded to the number of the injected dog and to the viral serotype used during this process. Next, the GFP-positive areas along the needle track in the slides containing caudate nucleus and thalamus (injection sites 1 and 2) were scanned at × 10 using a Leica DM RBE fluorescent microscope (Leica Microsystems, Wetzlar, Germany) equipped with a SPOT RTSE CCD camera (Diagnostic Instruments, Sterling Heights, MI, USA). Each dog had two injection sites evaluated for a total of four slides imaged per viral serotype. Mosaics of the scanned areas, containing as many as 128 images, were composed using iVision analytical software (BioVision Technologies, Exton, PA, USA), and measurements of maximum diameter and total GFP-positive area were made using this software. All images were taken under the same conditions with the same exposure times and a threshold established with identical 8-bit greyscale values.

### Immunofluorescence

The following primary antibodies were used for the co-labeling experiments in this study: chicken anti-GFP (Abcam #ab13970, Cambridge, MA, USA) diluted to 1:300; mouse anti-NeuN (Millipore #MAB377, Temecula, CA, USA) diluted to 1:400; rabbit anti-Olig2 (Millipore #AB9610) diluted to 1:100 and rabbit anti-GFAP (DakoCytomation #Z0334, Carpinteria, CA, USA) diluted to 1:400. Secondary antibodies used were AlexaFluor 488 goat anti-chicken, AlexaFluor 568 goat anti-mouse and AlexaFluor 568 goat anti-rabbit (Life Technologies, Carlsbad, CA, USA) all diluted to 1:400. Tissue sections were removed from a −80 °C freezer and allowed to dry for 1 h at room temperature and washed in PBS. Antigen retrieval was performed in 10 mM citric acid buffer pH6 using a Retriever 2100 autoclave (Prestige Medical Ltd., Coventry, UK). Primary antibodies were diluted pairwise in PBT (150 mM PBS/0.1% bovine serum albumin/0.2% Triton-X 100) and incubated at 4 °C for 15 h. The sections were then washed in several changes of PBS and secondary antibodies diluted pairwise in PBT and incubated at 37 °C for 40 min. Slides were washed in PBS and mounted in Vectashield/4',6-diamidino-2-phenylindole (#H-1200 Vector Laboratories, Burlingame, CA, USA).

### Statistics

Median and minimum and maximum were used to describe all data. The Kruskal–Wallis test was initially used to compare data between groups and if found significant (*P*<0.05), the Mann–Whitney test was used for pairwise comparisons to determine which specific groups were different (Stata for Mac 21.1, Stata Corporation, College Station, TX, USA). Significance was determined when *P*<0.05.

## Figures and Tables

**Table 1:** Naturally occurring genetic diseases of the canine CNS^1, 2, 3, 4, 5, 6, 7, 8, 9, 10, 11, 12, 13, 14, 15^

*Disease*	*Gene*	*Dog breeds affected*
Amyotrophic lateral sclerosis	*SOD1*	Boxer, German shepherd, Chesapeake Bay retriever, Standard poodle, Rhodesian ridgeback, Pembroke and Cardigan Welsh corgi, Bernese mountain, American eskimo, Golden retriever, Great Pyrenese, Kerry blue terrier, Pug, Shetland sheep dog, soft-coated Wheaton terrier, Wire fox terrier
Cerebellar ataxia	*SEL1L* *SPTBN2*	Finnish hound beagle
Juvenile epilepsy	*LGI2*	Lagotto Romagnolo
L-2-hydroxyglutaric aciduria	*L2HGDH*	Staffordshire bull terriers, Yorkshire terrier
		
Lysosomal storage diseases		
Ceroid lipofuscinosis	*CTSD* *CLN1 (PPT1)* *CLN2 (TPP1)* *CLN5* *CLN6* *CLN8* *ARSG (NCL-A4)* *ATP13A2*	American bulldogs Dachshund Dachshund Border collie Australian shepherd English/Irish setter American Staffordshire terriers Tibetan terrier
Fucosidosis	*FUCA-1*	English springer spaniel
Globoid cell leukodystrophy (Krabbe disease)	*GALC*	Cairn terrier dog, West Highland white terrier, Beagle, Blue tick hound, Irish setter, Australian Kelpie
Galactosialidosis	*CTSA*	Schipperke
Glucocerebrosidosis (Gaucher disease)	*GBA*	Sydney silky terrier
Glycogenosis type 1a	*G6PC*	Maltese dog
Glycogen storage disease II (Pompe Disease)	*GAA*	Lapland dog
Glycogenosis type IIIa	*AGL*	Curly-coated retriever
GM1 gangliosidosis	*GLB1*	Beagle mixbreed, Shiba inu, Springer spaniel, Portuguese water dog, English springer spaniel, Siberian husky
GM2 gangliosidosis (Tay–Sachs disease)	*HEX-A*	German short-haired pointer
GM2 gangliosidosis (Sandhoff disease)	HEX-B	Toy poodle, Japanese spaniel, Golden retriever, Silky terrier
Mucopolysaccharidosis I (Hurler, Scheie and Hurler/Scheie)	*IDUA*	Plotthound, Rottweiler, Afghan
Mucopolysaccharidosis II (Hunter syndrome)	*IDS*	Labrador retriever
Mucopolysaccharidosis III A (Sanfilippo A syndrome)	*SGSH*	Wire-haired dachshund, New Zealand huntaway
Mucopolysaccharidosis III B (Sanfilippo B syndrome)	*NAGLU*	Schipperke
Mucopolysaccharidosis VI (Maroteaux–Lamy syndrome)	*ARSB*	Miniature pinscher, Welsh corgi, miniature schnauzer, Chesapeake Bay retriever, Miniature poodle
Mucopolysaccharidosis VII (Sly disease)	*GUSB*	German shepherd, Brazilian terrier
Neuronal glycoproteinosis (Myoclonic epilepsy of Lafora)	*EPM2B (NHLRC1)*	Bassett hound, Beagle, Poodle, Wire-haired miniature dachshund
Sphingomyelinosis A and B (Niemann-Pick A and B diseases)	*SMPD1*	Miniature poodle
Narcolepsy	*HCRTR2*	Dachshund, Labrador retriever, Doberman
Neonatal cerebellar ataxia (Bandera's syndrome)	*GRM1*	Coton de Tulear
Neonatal encephalopathy	*ATF2*	Standard poodle
Neuroaxonal dystrophy	*MFN2*	Giant schnauzer
Pelizaeus–Merzbacher	*PLP*	Springer spaniel
Spongiform leukoencephalomyelopathy	*mtDNA*	Australian cattle dogs, Shetland sheep dogs
Thiamine deficiency encephalopathy	*SLC19A3*	Alaskan husky

Abbreviation: CNS, central nervous system.

**Table 2:** Semiquantitative evaluation of transduction of dog brain gray and white matters by four rAAV serotypes injected into the left cerebral hemisphere

*Serotype*	*AAV2/1*	*AAV2/1*	*AAV2/5*	*AAV2/5*	*AAV2/9*	*AAV2/9*	*AAV2/rh10*	*AAV2/rh10*
*Dog number*	*1*	*2*	*3*	*4*	*5*	*6*	*7*	*8*
Caudate nucleus	++	−	−	−	++++	+	++	−
Thalamus	++	+++	+	−	++++	+++	++++	+++
Cerebral cortex	+	++	+	−	++	++	−	++
Temporal lobe white matter	++	+++	+	+	++++	+++	+++	+++
Occipital lobe white matter	+	++	−	−	++	++	+++	++
Contralateral white matter	−	−	−	−	++	−	−	+

−, none.

+, sparse (<10 GFP-positive somata/ × 10 field; <5% GFP-positive axons/ × 10 field).

++, few (10–40 GFP-positive somata/ × 10 field; 5–10% GFP-positive axons/ × 10 field).

+++, moderate (40–100 GFP-positive somata/ × 10 field; 10–25% GFP-positive axons/ × 10 field).

++++, extensive (>100 GFP-positive somata/ × 10 field; >25% GFP × positive axons/ × 10 field).
